# Ultrabright Green-Emitting Nanoemulsions Based on Natural Lipids-BODIPY Conjugates

**DOI:** 10.3390/nano11030826

**Published:** 2021-03-23

**Authors:** Xinyue Wang, Sophie Bou, Andrey S. Klymchenko, Nicolas Anton, Mayeul Collot

**Affiliations:** 1 Faculté de Pharmacie d’Illkirch, Université de Strasbourg, CNRS, CAMB UMR 7199, F-67000 Strasbourg, France; ooxinyue21@live.com; 2 INSERM (French National Institute of Health and Medical Research), Université de Strasbourg, Regenerative Nanomedicine (RNM), FMTS, UMR 1260, F-67000 Strasbourg, France; 3 Faculté de Pharmacie d’Illkirch, Université de Strasbourg, CNRS, LPB 7021, F-67000 Strasbourg, France; s.bou@unistra.fr (S.B.); andrey.klymchenko@unistra.fr (A.S.K.)

**Keywords:** nanoemulsions, BODIPY, fluorescence, brightness, fluorescence correlation spectroscopy

## Abstract

Nanoemulsions (NEs) are water-dispersed oil droplets that constitute stealth biocompatible nanomaterials. NEs can reach an impressive degree of fluorescent brightness owing to their oily core that can encapsulate a large number of fluorophores on the condition the latter are sufficiently hydrophobic and oil-soluble. BODIPYs are among the brightest green emitting fluorophores and as neutral molecules possess high lipophilicity. Herein, we synthesized three different natural lipid-BODIPY conjugates by esterification of an acidic BODIPY by natural lipids, namely: α-tocopherol (vitamin E), cholesterol, and stearyl alcohol. The new BODIPY conjugates were characterized in solvents and oils before being encapsulated in NEs at various concentrations. The physical (size, stability over time, leakage) and photophysical properties (absorption and emission wavelength, brightness, photostability) are reported and showed that the nature of the lipid anchor and the nature of the oil used for emulsification greatly influence the properties of the bright NEs.

## 1. Introduction

Among nanomaterials, lipid nanoparticles draw a particular attention owing to the biocompatibility of their components [1,2]. Nanoemulsions (NEs) are nano-sized (20 to 300 nm) lipid-oil droplets stabilized with surfactants that found their applications in biology and medicine due to their stability and biocompatibility [3,4,5]. The oily core of NEs can serve as a drug reservoir to develop drug nanocarriers, [6] but can also encapsulate a great number of fluorescent dyes to obtain ultrabright fluorescent nanomaterials [7,8,9]. We recently reviewed dye-loaded NEs and their applications in bioimaging and nanomedicine where the required properties of the encapsulated dye were pointed out [9]. The latter must be highly lipophilic in order to reach high concentrations in the nano-droplet’s oil core, along with high stability to prevent premature leakage of the dye [8,9,10]. Moreover, they should bear relatively bulky groups or bulky counterions in order to prevent their aggregation-caused quenching within the oil core [7,9,11].

**Figure 1:**
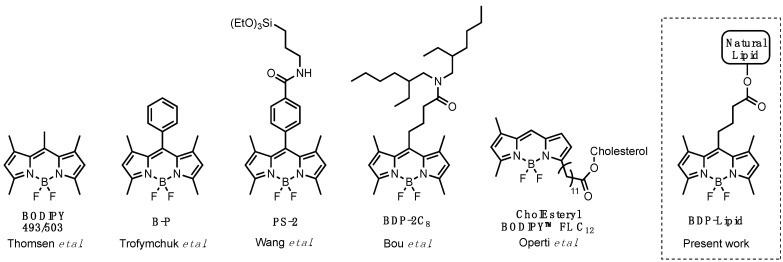
Green emitting BODIPYs used for dye-loaded nanomaterials.

In bioimaging the “green channel” is generally the most accessible due to the widely spread argon laser line at 488 nm as well as the wide use of GFP-labeled proteins in biological studies. Therefore, bright green emitting NEs would be a valuable tool for studying their potential application in biology and medicine using fluorescence bioimaging. Cationic cyanine DiO [12,13] and perylene-based dye [14] were already used to obtain green emitting NEs but suffer from low hydrophobicity due to a cationic nature and limited photophysical properties, respectively. Conversely, BODIPY (4,4-Difluoro-4-bora-3a,4a-diaza-*s*-indacene) seems perfectly adapted for this purpose, as it is a noncharged green-emitting fluorophore. Moreover, it has received considerable interest in bioimaging applications owing to its desirable properties, including narrow emission band, high quantum yield and molar extinction coefficient (ε), making it a bright-green fluorophore [15,16,17]. Surprisingly, green BODIPYs have never been used as fluorescent cargos for NEs. However, different green BODIPYs successfully served to load nanomaterials (Figure 1), including BODIPY 493/503 for poly(D,L-lactic acid) based nanoparticles [18], P-B and CholEsteryl BODIPY FL C12 for poly(lactic-co-glycolic acid) (PLGA) [19,20], PS-2 for silica NPs [21], and BDP-2C_8_ for oil/polymer hybrid NPs [22]. Although the latter possesses enhanced hydrophobicity due to hydrophobic branched aliphatic chains, its biocompatibility remained limited and was only used for in vitro applications. Conversely, the use of commercially available CholEsteryl BODIPY FL C_12_ constitutes an interesting approach as it is composed of cholesterol, a natural nontoxic lipid. 

In this work we thus synthesized three natural lipid BODIPY conjugates bearing a biocleavable ester bond [21] and studied their ability to provide ultrabright and stable NEs for bioimaging applications. To ensure the high lipophilicity of the conjugates and their biocompatibility, we chose three natural lipids with high hydrophobicity, bearing a hydroxyl group for esterification. Cholesterol was chosen as a cost-effective natural and nontoxic sterol. α-tocopherol is a liposoluble vitamin (vitamin E) that is found in vegetal oils. Finally, stearyl alcohol was chosen as an aliphatic alcohol with a C_18_ hydrocarbon chain that was shown to have a very low toxicity (LD_50_ > 5000 mg/kg in rat) [23]. NEs composed of middle chain triglycerides (MCT) or vitamin E acetate (VEA, also known as tocopherol acetate) were loaded with the conjugates at various weight percentage (wt %) and their properties were studied. This study showed that both the lipid anchor and the nature of the NEs’ oily core played important roles in the design of ultrabright NEs.

## 2. Materials and Methods

### 2.1. Synthesis

All starting materials for synthesis were purchased from Sigma-Aldrich, TCI or Alfa Aesar and were used as received unless stated otherwise. Labrafac WL 1349 (medium chain triglycerides, MCT) was obtained from Gattefossé (Saint-Priest, France), Vitamin E Acetate (VEA) was purchased from Tokyo Chemical Industry (Tokyo, Japan), Kolliphor ELP was from BASF (Ludwigshafen, Germany). NMR spectra were recorded in deuterated chloroform (CDCl_3_) at a concentration of 15 mg.mL^−1^ on a Bruker Avance III 400 MHz spectrometer (Rheinstetten, Germany). Mass spectra were obtained using an Agilent Q-TOF 6520 mass spectrometer (Agilent Technologies, Santa Clara, CA, USA); the samples were submitted in dichloromethane at a concentration of 0.1 mg.mL^−1^. The protocol for synthesis of all new compounds as well as NMR and mass spectra can be found in the Supporting Information.

### 2.2. Spectroscopy

Absorption and emission spectra were recorded on a Varian Cary 4000 HP Scan ultraviolet-visible spectrophotometer (Agilent, Santa Clara, CA, USA) and a FluoroMax-4 spectrofluorometer (Horiba Jobin Yvon, Kyoto, Japan) equipped with a thermostated cell compartment, respectively. For standard recording of fluorescence spectra, the emission was collected 10 nm after the excitation wavelength. Concentration used for spectroscopic study was set as 1 μM for each dye. The molecular extinction coefficients were obtained from a stock solution that was obtained by dissolving at least 5 mg of BODIPY conjugate in dioxane. The quantum yields were determined by comparison with fluorescein in 0.1 M NaOH (QY = 0.95) as reference [24] using the following equation:

(1) $$\mathrm{QY}={\mathrm{QY}}_{R}\times \;\frac{I\times {\mathrm{OD}}_{R}\times n^{2}}{I_{R}\times \mathrm{OD}\times {\;n}_{R}^{2}}$$

where QY is the quantum yield, I is the integrated fluorescence intensity, n is the refractive index, and OD is the optical density at the excitation wavelength. R represents the reference.

### 2.3. Solubility Test in Oils

Saturated solutions of dyes in oils were prepared by weighting 12 mg of dye to which was added 100 mg of oil at 80 °C (due to the high viscosity of oil) under stirring. The solution was cooled down to room temperature before being centrifuged at 10,000 rpm for 1 min. 5 μL of the supernatant was taken and diluted in dioxane for an absorbance measurement. Solubility of individual dye was calculated by the following equation:

(2) $$S\left(\mathrm{wt}\;\%\right)=\frac{\mathrm{OD}\times f\times 1\;\mathrm{mL}\times \mathrm{MW}}{\varepsilon \times 100\;\mathrm{mg}}\times 100$$

where OD is the measured optical density, f is the dilution factor (varies from dye to dye), 1 mL is the final volume after dilution used for the absorption test, MW is the molar weight of each dye, ε is the molar extinction coefficient of each dye in dioxane, and 100 mg is the weight of saturated dye−oil solution.

### 2.4. Formulation and Characterization of Nanoemulsions

Nanoemulsions (NEs) were formulated by spontaneous emulsification method, as described previously [25]. Briefly, the BODIPY conjugates were first dissolved in oil (100 mg) and mixed with surfactant (Kolliphor ELP, 100 mg) at a set surfactant-to-oil ratio (SOR = 1), with a temperature higher than the cloud point of the nonionic surfactant (i.e., 90 °C). Then, 300 μL of hot Milli-Q water (90 °C) was quickly added to the oil mixture under stirring to induce the spontaneous emulsification. The NEs were then homogenized (vortex) for 1 min followed by thermo-mixing at room temperature. 

### 2.5. Characterization and Stability of Nanoemulsions

The obtained NEs were characterized by dynamic light scattering (DLS) Malvern Zetasizer ZSP (Malvern, UK) with a laser operating at 633 nm, and the temperature was maintained at 25 °C. The average size and polydispersity index (PDI) of the NEs were measured three times from three independent formulations. The formulations were stored for two weeks at room temperature for a stability study, and size and PDI were measured.

### 2.6. Photostability Test

NEs with 1% dye loading were chosen for the photostability test. All the NEs were diluted to keep the dye concentration at 1 μM in the final solutions. To make sure that all the NEs were excited at the same extinction coefficient (ε = 40,000 M^−1^.cm^−1^), 470 nm was chosen as the excitation wavelength (slit opening was set to 16 nm). The emission signal was monitored at 600 nm for all fluorophores. The measurement was conducted over 1 h. A relative fluorescence intensity (%) was normalized to the maximum intensity at the starting point. For the loading dependency experiment, the same method was used except that the emission wavelength was set to 500 nm to increase the photodamage.

### 2.7. Fluorescence Correlation Spectroscopy (FCS)

FCS measurements were performed on a home-built confocal set-up based on a Nikon inverted microscope with a Nikon 60× 1.2NA (Tokyo, Japan) water immersion objective. Excitation was provided by a cw laser diode (488 nm, Oxxius, Lannion, France) and photons were detected through a bandpass 525/50 nm filter (Semrock, Rochester, NY, USA) with a fibered avalanche photodiode (APD SPCM-AQR-14-FC, PerkinElmer, Rodgau, Germany) connected to an online hardware correlator (ALV7000-USB, ALV GmbH, Hessen, Germany). The typical acquisition time was 5 min (10 × 30 s) with an excitation power of 0.05 mW at the sample level. The data were analyzed using PyCorrFit software [26]. The FCS study used 100 times diluted NEs for the size measurement. Before measurement, another 100-fold dilution was performed for all the samples, respectively, in PBS and 10% FBS (fetal bovine serum) + 90% PBS solutions. Solution of fluorescein (50 nM in 0.1 M NaOH in water) was used as a reference. 200 μL of each sample was placed in a 96-well plate.

## 3. Results

### 3.1. Synthesis of the Lipid-BODIPY Conjugates

**Scheme 1:**
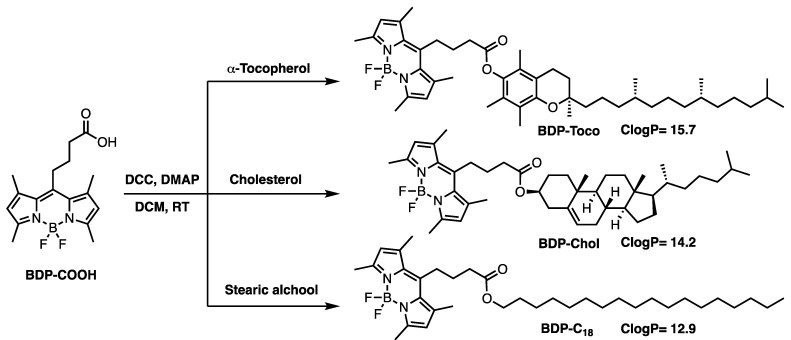
Synthesis of three natural lipids-BODIPY conjugates from: α-tocopherol: BDP-Toco; cholesterol: BDP-Chol and Stearyl alcohol: BDP-C_18_.

Although hydrophobic lipid BODIPY esters have already been developed, their accessibility or photochemical properties remain limited [27,28]. Moreover, we decided to use the same BODIPY platform to investigate the effect of the lipid anchor. BDP-COOH was thus chosen as a bright BODIPY that bears a carboxylic acid-ended aliphatic chain at the *meso* position [29,30]. The natural lipid-BODIPY conjugates were obtained by esterification coupling between BDP-COOH and the three different natural lipids using DCC, DMAP (Scheme 1). After purification the products were characterized by ^1^H and ^13^C NMR as well as mass spectroscopy (see supplementary information). The calculated logP (ClogP obtained by Chemdraw, PerkinElmer, Rodgau, Germany) of the BODIPY conjugates were all above 12 (Scheme 1), which denotes a high hydrophobic nature that should ensure a good solubility in oils and prevent detrimental dye leakage (vide infra).

### 3.2. Photophysical Studies of BODIPY Conjugates

**Figure 2:**
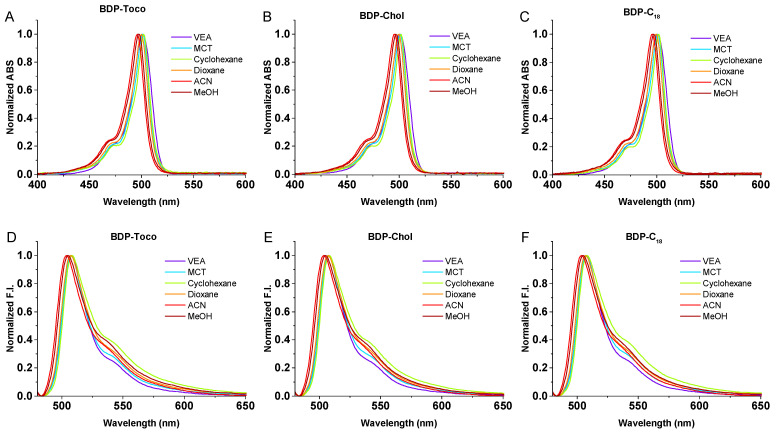
Normalized absorption (**top**) and emission (**bottom**) spectra of BDP-Toco (**A**,**D**), BDP-Chol (**B**,**E**) and BDP-C_18_ (**C**,**F**) in various solvents and oils at 1 μM. Excitation wavelength was 470 nm.

The photophysical properties of the BODIPY conjugates were studied in various organic solvents with increasing polarity from cyclohexane to methanol as well as in lipid oils compatible with spontaneous nanoemulsification process [8,31] namely, medium chain triglycerides and vitamin E acetate. The results showed that the BODIPY conjugates displayed similar photophysical properties regardless of the solubilizing medium (Figure 2). As expected, the green emitting BODIPY conjugates exhibited maximum absorption and emission wavelengths at 500 ± 2 nm and 506 ± 2 nm, respectively with very narrow peaks (see FWHM values in Table 1). Additionally, owing to their high molecular extinction coefficients (ε) combined to their high quantum yields (φ), the conjugates showed high brightness (ε × φ) up to 80,100 M^−1^.cm^−1^. Importantly, notable differences were observed between the two lipid oils. Although the BDPs displayed identical maximum absorption and emission wavelengths in both oils, the absorption peak slightly broadens in VEA (FWHM from 18 to 21 nm), probably denoting a difference in solvatation or interaction for the two oils. Compared to MCT, BDPs in VEA systematically displayed lower ε, which was compensated with higher quantum yields in the case of BDP-C_18_ and BDP-Toco, reaching up to 0.98 quantum yield for the latter (Table 1). Overall, the BODIPY conjugates exhibited high brightness values in the lipid oil, comparable to those obtained in hydrophobic cyclohexane, thus constituting a promising combination for the development of bright green emitting NEs. The properties of the dyes were also assessed in water. As expected, in water the hydrophobic conjugates formed aggregates, which were identified by the broadening of their absorption peak, the significant red shift in both absorption and emission spectra (Figure S13) as well as the drop of the quantum yield due to aggregation-caused quenching (Table 1) [32]. Interestingly, BDP-C_18_ and BDP-cholesterol formed relatively bright orange emitting aggregates in water (λ_Em_ = 570–576 nm) with quantum yield reaching 0.13 and 0.18, respectively (Table 1). Conversely, BDP-Toco formed low emitting aggregates (φ = 0.04) denoting the influence of the lipid anchor on the aggregation pattern of the conjugates. Overall, BDP-Toco presented several advantages for dye-loaded NEs owing to its superior brightness in oil and its poorly emissive aggregates in water.

### 3.3. Solubility of BDP Conjugates in Oils

The solubility of the encapsulated dye in NEs is of prior importance as it determines the maximum loading capacity of the NE droplets, and hence condition the maximum brightness that the NEs can reach. We thus assessed the maximum solubility of our BDP conjugates in MCT and VEA that served for nanoemulsification [8,25]. As illustrated in Table 2, the three BDP conjugates showed different solubility. On the one hand, BDP-Chol and BDP-C_18_ that are solids, showed maximum solubility in oils of 3.3 wt % (BDP-Chol in VEA), which is comparable with other dyes used for loading NEs [8,10]. On the other hand, BDP-Toco, which is an oil at room temperature, was found to be miscible with both MCT and VEA at any ratio. To avoid aggregation-caused quenching (ACQ) that occurs at very high wt %, the maximum loading was limited to 12 wt % for our study. These differences could be assigned to the difference of crystallinity brought by the lipid anchor. Indeed, whereas α-tocopherol is liquid at room temperature (melting point ∼3 °C), cholesterol and stearyl alcohol are solids (melting point = 148 °C and 60 °C, respectively).

### 3.4. BDP-Loaded Nanoemulsions

**Figure 3:**
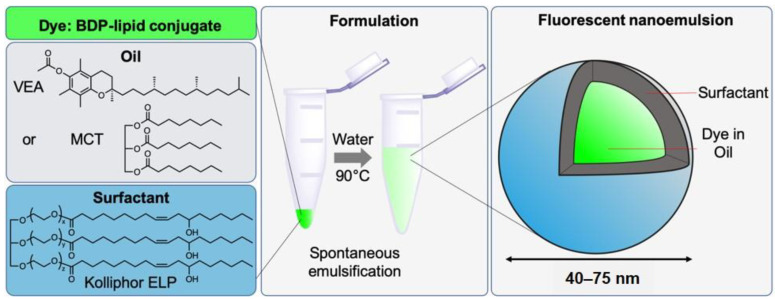
Composition and formulation of bright fluorescent NEs based on BDP-lipid conjugates.

**Figure 4:**
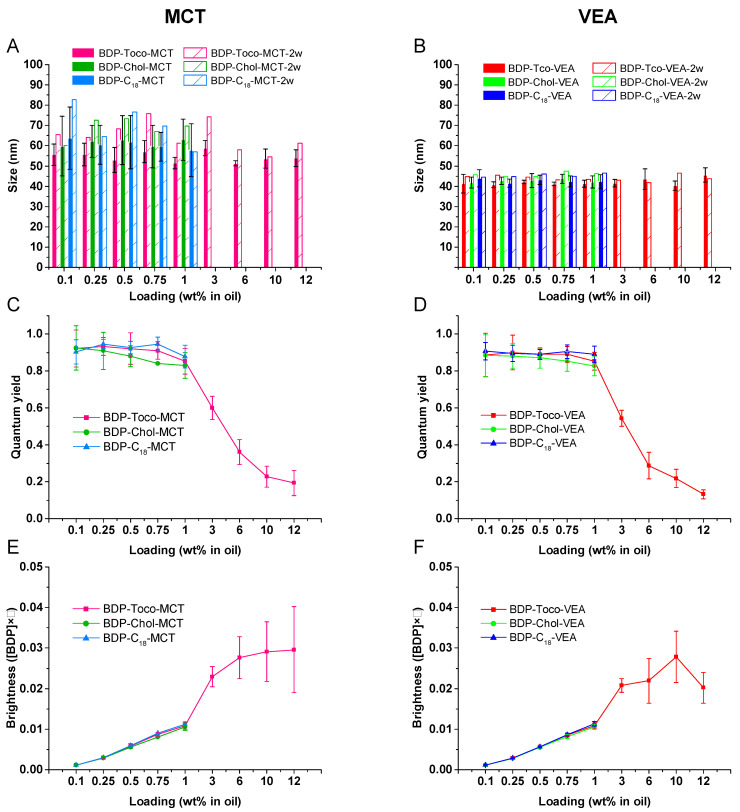
Properties of the BDP-loaded NEs in MCT (**left**) and in VEA (**right**). (**A**,**B**) Average size measured by DLS, solid bars are freshly prepared NEs and dashed bars are NEs after two weeks (indicated with the notation “−2w” in the insets). PDI averaged between 0.13 for MCT and 0.05 for VEA based NEs. Quantum yields of BDP-loaded NEs upon wt % increase (**C**,**D**) and their corresponding relative brightness (**E**,**F**), expressed as φ ×[BDP] (unit: Molar), taking into account the molecular weight of the conjugates and regardless of the NEs’ size.

Considering the interesting photophysical properties of the BODIPY conjugates in MCT and VEA, namely high solubility, narrow emission bands and high brightness, these oils were used for the formulation of fluorescent NEs with increasing loading of BODIPY conjugates up to 1 wt % for BDP-Chol and BDP-C_18_ and up to 12 wt % for BDP-Toco. To this endeavor, spontaneous nanoemulsification with nonionic PEGylated surfactant (Kolliphor ELP) as a water/oil interface stabilizer was employed (Figure 3). Once the NEs obtained, their size distributions were determined by DLS. The results showed a significant difference between the two oils. Whereas the sizes of NEs with MCT fluctuated between 50–75 nm with PDI values around 0.1 (Figure 4A), those obtained from VEA displayed a narrow range of size of 40–50 nm with polydispersity index (PDI) values around 0.05 regardless of the dye loading (Figure 4B). The stability over the time of the formed NEs was assessed by measuring their size after two weeks. The results, illustrated in Figure 4A,B, showed that the sizes of MCT based-NEs were increased by ~10 nm in average, whereas the sizes of VEA based-NEs only slightly increased (<5 nm). These results show that, compared to MCT, the use of VEA led to more stable and monodisperse NEs, possibly due to the higher viscosity and hydrophobicity of VEA oil that prevent Ostwald ripening of the suspension of NEs droplets [33].

In a second step, the quantum yields of the different NEs were measured. The results showed that the NEs conserved high quantum yields (~0.9) even at 1 wt % loading (corresponding to molar concentrations >10^−2^ M of dye in oil), regardless of the used lipid oils and the BODIPY-lipid conjugates (Figure 4C,D). Indeed, at 1 wt % loading none of the BODIPY conjugates displayed any sign of aggregation as proven by their absorption and emission spectra (Figure S14). In the case of BDP-Toco that could be solubilized up to 12 wt % in oils, a decrease of quantum yield was observed after 1 wt % loading, which was clearly assigned to the ACQ phenomenon. This was confirmed by both absorption and emission spectra broadening along with a red shift in the emission (Figure S15) [32]. Despite this phenomenon, it was noteworthy that at the very high loading percentage of 12 wt %, the quantum yield value was not negligible (~0.2). By plotting the relative brightness of NEs (φ × molar concentration of the dye in oil), regardless of their size and considering the molecular weight of the conjugates, all samples showed very similar results (see Figure 4E,F) for dye loadings below 1 wt %. However, NEs loaded with BDP-Toco exhibited high levels of brightness reaching a plateau at 10 wt % in MCT, owing to its high solubility in oil. The achieved maximum brightness at 10 wt % in VEA was ~3-fold higher than that obtained with the other BODIPY conjugates at 1 wt % loading. Considering these 10 wt % VEA-based NEs loaded with BDP-Toco with a diameter size of ~40 nm (see Figure 4B), we estimated an impressive individual NEs’ brightness value of ~45.10^6^ M^−1^.cm^−1^ (calculations can be found in the supplementary information). As a comparison, this value corresponds to ~630 fluorescein dyes (dianionic form with ε = 76,900 mol^−1^.cm^−1^ and φ = 0.93) [34].

### 3.5. Dye Leakage

As discussed in the introduction, the dye encapsulated in NEs should not leak out. Indeed, dye leakage leads to a loss of NEs’ brightness as well as an increase of the background noise in bioimaging and labeling of the hydrophobic compartments such as membranes or proteins [9]. We thus assessed the dye leakage by fluorescence correlation spectroscopy (FCS). FCS is a powerful spectroscopic technique providing simultaneously information on size, concentration, and brightness of fluorescent nanomaterials by measuring the diffusion of fluorescent species in a focal volume [10,35]. In this study, measurements were conducted in PBS alone, and in PBS supplemented with 10% fetal bovine serum (FBS) as the biomolecules that compose FBS act as dye acceptors that can be found in biological environment.

Results presented in Table 3, first showed that 1 wt % loaded NEs formulated in PBS possess similar size compared to those formulated in water and measured by DLS (Figure 4A,B). Then, the dye leakage can be assessed by comparing the sizes and the concentrations of the NEs obtained in PBS and PBS-10% FBS. Indeed, in case of dye leakage the number of fluorescent species increases in the medium, leading to an increase of their concentration [10]. The results showed that the concentrations of fluorescent species remained unchanged in the presence of 10% FBS regardless of the dye or oil used, indicating the absence of dye leakage at 1 wt % loading.

Moreover, their size also remained stable, showing the stealth of the NEs, where PEGylated shell probably prevents nonspecific binding of serum proteins. Finally, FCS was performed on the highly loaded BDP-Toco NEs (from 1 to 12 wt %). Surprisingly, at high loadings the size and concentration of fluorescent species only slightly increased in the presence of FBS, denoting a strong retention of the BDP-Toco dye in the NEs even at loading as high as 12 wt % (Table 4). Additionally, it was interesting to note that upon increase of dye loading, the concentration of fluorescent species significantly decreased (for both media PBS and PBS-10% FBS). This observation was assigned to the decrease of photostability upon increase of dye loading, which is in line with our recent study with BODIPY loaded polymeric NPs, where we showed that concentrating BODIPYs could lead to bright nanomaterials but with high photobleaching rate [32].

### 3.6. Photostability

**Figure 5:**
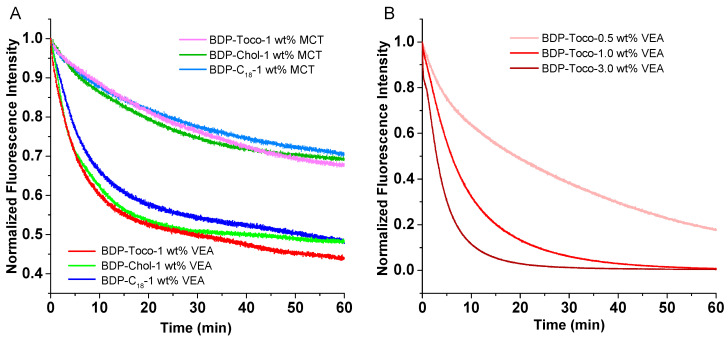
(**A**) Photostability of 1 wt % dye-loaded NEs under continuous illumination. (**B**) Photostability of BDP-Toco loaded NEs with increase in loading wt % under continuous illumination. λ_Ex_ was 470 nm for A and 500 nm for B. The fluorescence was monitored at 600 nm.

For bioimaging purposes, the photostability is of prior importance as it will determine the ability to efficiently track the fluorescent nanomaterial over the time. We thus assessed the photostability of both MCT and VEA based NEs under continuous irradiation at 470 nm over one hour (Figure 5A). For a fair comparison, 1 wt % loading was chosen as it is the highest common concentration (lower limit: BDP-Chol, 1 wt % in MCT. Surprisingly the results showed that, unlike the lipid anchor of the BODIPY conjugates, the nature of the NE’s oil greatly influenced the photostability. Indeed, after 1 h of continuous irradiation VEA-based NEs lost half of their fluorescence intensity whereas MCT-based ones lost only 30%. This difference could be explained by the fact that VEA itself is photosensitive [36,37]. Therefore, under light irradiation VEA can undergo a photolysis that generates radicals, thus leading to degradation of the surrounding dyes. To confirm our observations during FCS experiment, a photostability assay was performed on VEA-based NEs loaded with an increasing loading of BDP-Toco from 0.5 wt % to 3 wt % and at the maximum excitation wavelength (λ_Ex_ = 500 nm). The results clearly showed that a higher BDP loading led to NEs with lowered photostability (Figure 5B).

## 4. Conclusions

The development of novel fluorescent nanoprobes with remarkable optical properties, suitable particle size, high water dispersibility and good biocompatibility are highly desirable for applications in the biomedical field. In this work, we introduced three BODIPY conjugates with natural lipid anchors, namely tocopherol (vitamin E), cholesterol and stearyl alcohol. Those three dyes exhibit high molar extinction coefficient, quantum yield and solubility in oils, making them good candidates for encapsulation in NEs. As expected, bright fluorescent nanoemulsions were obtained by spontaneous emulsification with small sizes ranging from 40 to 75 nm, good polydispersity index, and without obvious leakage in the presence of FBS. Among the BODIPY conjugates, BDP-Toco is an oil with high miscibility in MCT and VEA. This specific property allowed to obtain NEs with high dye loading percentage and thus ultrabright NEs due to relatively low aggregation-caused quenching (ACQ) at high concentration of BDP. The combination of tocopherol acetate (VEA) and tocopherol BDP ester (BDP-Toco) constituted an effective match as it led to small (~40 nm), physically stable, hermetic (no leakage) and ultrabright (brightness = 45.10^6^ M^−1^.cm^−1^) NEs. However, FCS and photostability assays indicated that highly loaded and bright NEs suffer from an important photo instability, probably due to the tendency of the BODIPY fluorophore to undergo destructive photochemical reactions when concentrated, thus limiting its use in NEs tracking. Previously developed fluorescent NEs generally operate in the red and near-infrared regions, [7,8,10,11] while examples of green emitting NEs are rare [14]. In the present work, owing to the rational design of BODIPY dyes, we achieved, to the best of our knowledge, the brightest green emitting NEs reported to date. This was possible notably because BDP-Toco was missile with oil and thus showed better solubility than NR668, [10] dioxaborines [8] and perylene derivatives, [14] while being comparable to F888 [10] and DiI-TPB [11]. In conclusion, this study emphasizes that tocopherol could be generalized as an efficient natural lipid anchor to efficiently load NEs with fluorophores as well as other biomolecules for potential drug delivery or therapeutics applications.

## Figures and Tables

**Table 1:** Photophysical properties of BODIPY conjugates in various solvents and oils at 1 μM.

BDP-Toco								
Solvent	λ_Abs_ (nm)	ε (M^−1^ cm^−1^)	FWHM Abs (nm)	λ_Em_ (nm)	FWHM Em (nm)	Stokes Shift (nm)	φ ^a^ (QY)	Brightness ^b^ (M^−1^ cm^−1^)
VEA	501	74,800	21	508	24	7	0.98	73,400
MCT	501	82,400	18	508	24	7	0.92	75,600
Cyclohexane	502	90,900	20	508	28	6	0.88	80,100
Dioxane	500	83,100	18	507	26	7	0.83	68,800
ACN	496	75,100	19	504	27	8	0.75	56,700
MeOH	498	82,900	19	505	29	7	0.78	64,400
Water	507	32,800	59	699	N/A ^c^	N/A ^c^	0.04	1250
**BDP-Chol**								
**Solvent**	**λ_Abs_** **(nm)**	**ε (M^−1^ cm^−1^)**	**FWHM Abs (nm)**	**λ_Em_** **(nm)**	**FWHM Em (nm)**	**Stokes Shift (nm)**	**φ ^a^ (QY)**	**Brightness** **^b^ (M^−1^ cm^−1^)**
VEA	501	70,000	21	507	24	6	0.89	62,000
MCT	501	73,500	18	507	24	6	0.92	67,700
Cyclohexane	502	77,000	16	508	28	6	0.81	62,700
Dioxane	500	75,100	18	507	24	7	0.76	56,900
ACN	496	68,900	19	504	28	8	0.67	46,500
MeOH	497	70,800	16	505	29	8	0.73	51,800
Water	507	33,800	56	570	N/A ^c^	N/A ^c^	0.18	6000
**BDP-C_18_**								
**Solvent**	**λ_Abs_** **(nm)**	**ε (M^−1^ cm^−1^)**	**FWHM Abs (nm)**	**λ_Em_** **(nm)**	**FWHM Em (nm)**	**Stokes shift (nm)**	**φ ^a^ (QY)**	**Brightness** **^b^ (M^−1^ cm^−1^)**
VEA	501	64,600	21	508	24	7	0.99	64,100
MCT	501	73,900	18	507	25	6	0.95	70,400
Cyclohexane	502	81,200	16	508	29	6	0.82	66,700
Dioxane	499	74,400	18	507	27	8	0.8	59,100
ACN	496	66,800	19	504	28	8	0.68	45,600
MeOH	497	68,900	19	505	29	8	0.74	50,700
Water	503	28,600	57	576	N/A ^c^	N/A ^c^	0.13	3800

^a^ Quantum yields were measured using Fluorescein in 0.1 M NaOH (φ = 0.95) as a reference [24]. ^b^ Brightness is QY × ε. ^c^ Very broad emission spectrum (Figure S13). N/A: not applicable.

**Table 2:** Solubility of three BODIPY conjugates in oils (at R.T.).

Oil	BDP-Toco	BDP-Chol	BDP-C_18_
MCT	>12 wt %	1.2 wt %	2.9 wt %
VEA	>12 wt %	3.3 wt %	2.7 wt %

**Table 3:** Assessment of dye leakage from the 1 wt % dye-loaded NEs by Fluorescence Correlation Spectroscopy (FCS).

BDP	Oil	Medium	Size (nm)	Concentration (nM)	BDP	Oil	Medium	Size (nm)	Concentration (nM)	Concentration Ratio (FBS/PBS)
**BDP-Toco**	MCT	PBS	64	5.84	**BDP-Toco**	MCT	FBS	62	4.79	0.82
**BDP-Chol**	MCT	PBS	86	2.83	**BDP-Chol**	MCT	FBS	84	2.97	1.05
**BDP-C_18_**	MCT	PBS	79	1.83	**BDP-C_18_**	MCT	FBS	78	2.13	1.16
**BDP-Toco**	VEA	PBS	45	7.63	**BDP-Toco**	VEA	FBS	47	9.32	1.22
**BDP-Chol**	VEA	PBS	50	8.18	**BDP-Chol**	VEA	FBS	51	9.08	1.11
**BDP-C_18_**	VEA	PBS	51	5.73	**BDP-C_18_**	VEA	FBS	53	6.93	1.21

**Table 4:** Assessment of the dye leakage by Fluorescence Correlation Spectroscopy (FCS).

BDP-Toco Loading	Oil	Medium	Size (nm)	Concentration (nM)	Concentration Ratio (FBS/PBS)
1 wt %	MCT	PBS/FBS	64/62	5.84/4.79	0.82
3 wt %	MCT	PBS/FBS	60/63	1.48/1.68	1.14
6 wt %	MCT	PBS/FBS	56/61	0.75/1.35	1.80
10 wt %	MCT	PBS/FBS	64/74	0.46/0.97	2.11
12 wt %	MCT	PBS/FBS	59/73	0.47/0.85	1.81
**BDP-Toco Loading**	**Oil**	**Medium**	**Size (nm)**	**Concentration (nM)**	**Concentration Ratio (FBS/PBS)**
1 wt %	VEA	PBS/FBS	45/47	7.63/9.32	1.22
3 wt %	VEA	PBS/FBS	46/47	2.43/3.55	1.46
6 wt %	VEA	PBS/FBS	42/43	1.32/2.93	2.22
10 wt %	VEA	PBS/FBS	47/46	1.27/2.36	1.86
12 wt %	VEA	PBS/FBS	52/53	1.05/1.77	1.69

## Data Availability

The data presented in this study are available on request from the corresponding author.”

## Supplementary Materials

The following are available online at https://www.mdpi.com/2079-4991/11/3/826/s1, ^1^H, ^13^C NMR and high-resolution mass spectra, Figures S1–S12, Supplementary absorption and emission spectra: Figures S13–S15.
