# Supplementary material for: Ultrabright Green-Emitting Nanoemulsions Based on Natural Lipids-BODIPY Conjugates
Source: Nanomaterials (Basel). 2021 Mar 23;11(3):826. doi: 10.3390/nano11030826 (PMC8005018; doi:10.3390/nano11030826)
Supplement: Supplementary file 1 [file nanomaterials-11-00826-s001.pdf]

Supplementary Material

Ultrabright Green-Emitting Nanoemulsions Based on Natural Lipids-BODIPY Conjugates

Xinyue Wang [1,2,†], Sophie Bou [3,†], Andrey S. Klymchenko [3], Nicolas Anton [1,2,*] and Mayeul Collot [3,*]

[1] Faculté de pharmacie d'Illkirch, Université de Strasbourg, CNRS, CAMB UMR 7199, F-67000 Strasbourg, France; ooxinyue21@live.com
[2] INSERM (French National Institute of Health and Medical Research), UMR 1260, Regenerative Nanomedicine (RNM), FMTS, Université de Strasbourg, F-67000 Strasbourg, France
[3] Faculté de pharmacie d'Illkirch,Université de Strasbourg, CNRS, LPB 7021, F-67000 Strasbourg, France; s.bou@unistra.fr (S.B.);
andrey.klymchenko@unistra.fr (A.S.K.)
* Correspondence: nanton@unistra.fr (N.A.); mayeul.collot@unistra.fr (M.C.)
† Contributed equally to this work

1. Synthesis of the natural lipid BDP conjugates

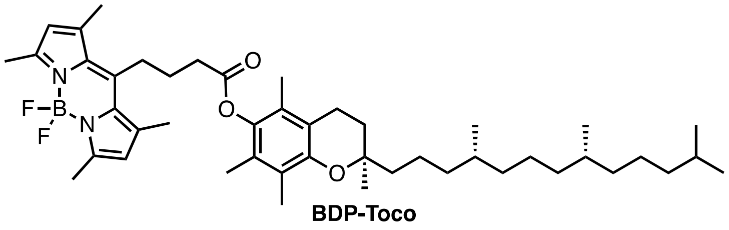

Figure S1. BDP-Toco

To a solution of BDP-COOH (100 mg, 0.299 mmol, 1 eq) in DCM (3 mL) under Ar atm, α-Tocopherol (193 mg, 0.449 mmol, 1.5 eq) was added, followed by DCC (74 mg, 0.359 mmol, 1.2 eq) and DMAP (7.3 mg, 0.060 mmol, 0.2 eq). The reaction mixture was allowed to stir for 0.5 h. Then, the product was extracted with HCl (1 M) and washed two times with saturated Na_2CO_3. The organic phase was dried over anhydrous $MgSO_4$, filtrated and concentrated. The crude was purified by chromatography column on silica gel using DCM/Heptane (1:1) to obtain 175 mg of **BCP-Toco** (Yield = 78%) as an orange oil. Rf = 0.55 (DCM/Heptane, 1:1). ^{1}H-NMR (400 MHz, $CDCl_3$): δ 6.11 (s, 2H, H-β BODIPY), 3.12 (dt, *J* = 7.8, 4.3 Hz, 2H, CH_2-CO), 2.85 (t, *J* = 7.1 Hz, 2H, CH_2), 2.65 (t, *J* = 6.7 Hz, 2H, CH_2), 2.59 (s, 6H, 2 CH_3 BDP), 2.50 (s, 6H, 2 CH_3 BDP), 2.19-2.14 (m, 7H, 2 CH_2, CH_3 Ar Toco), 2.08 (s, 3H, CH_3 Ar Toco), 2.03 (s, 3H, CH_3 Ar Toco), 1.85 (m, 3H), 1.63–1.15 (m, 30H, CH, CH_2 Toco), 0.94 (d, *J* = 6.8 Hz, 15H, 5 CH_3 Toco). 13-C NMR (126 MHz; CDCl3): δ 171.3, 154.2, 149.6, 144.9, 140.6, 140.4, 131.5, 126.5, 124.8, 123.2, 121.8, 117.5, 75.1, 39.4, 37.52, 37.45, 37.42, 37.35, 34.0, 32.85, 32.83, 32.74, 28.0, 27.7, 26.8, 24.89, 24.88, 24.5, 22.81, 22.71, 21.1, 20.7, 19.83, 19.77, 19.74, 19.71, 19.68, 16.5, 14.5, 13.1, 12.3, 11.9. HRMS (ES$^+$), calcd for $C_{46}H_{69}BF_2N_2O_3Na$ $[M+Na]^+$ 769.5267, found 769.5273.

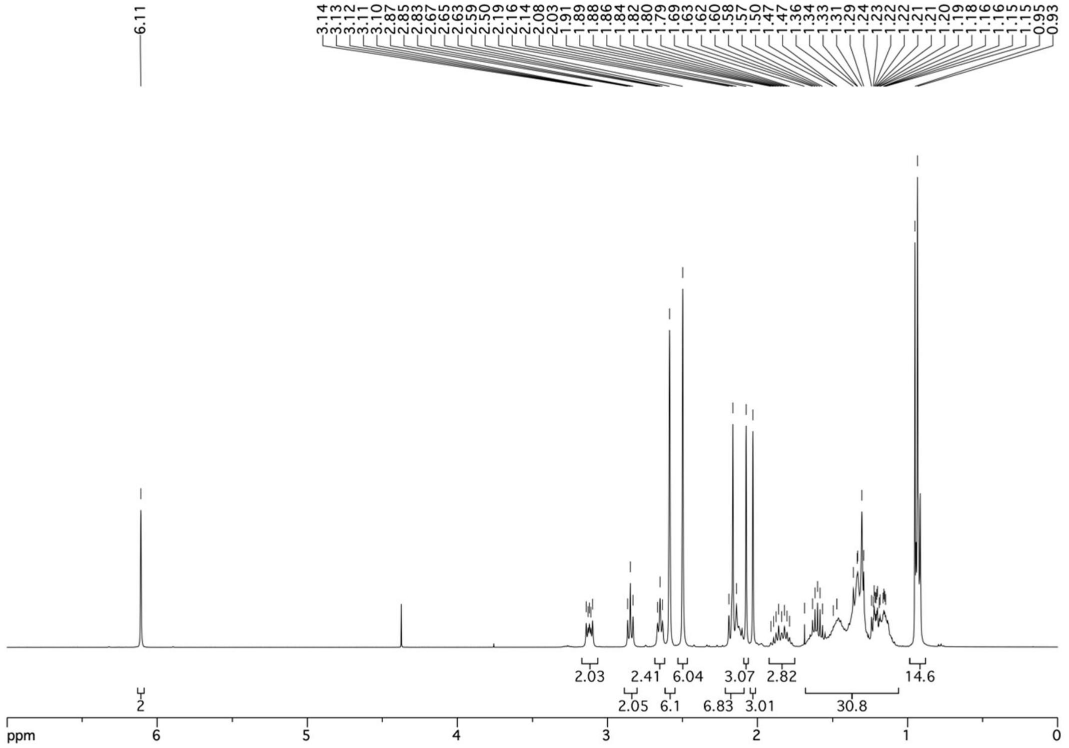

Figure S2. ^{1}H NMR spectrum of BDP-Toco.

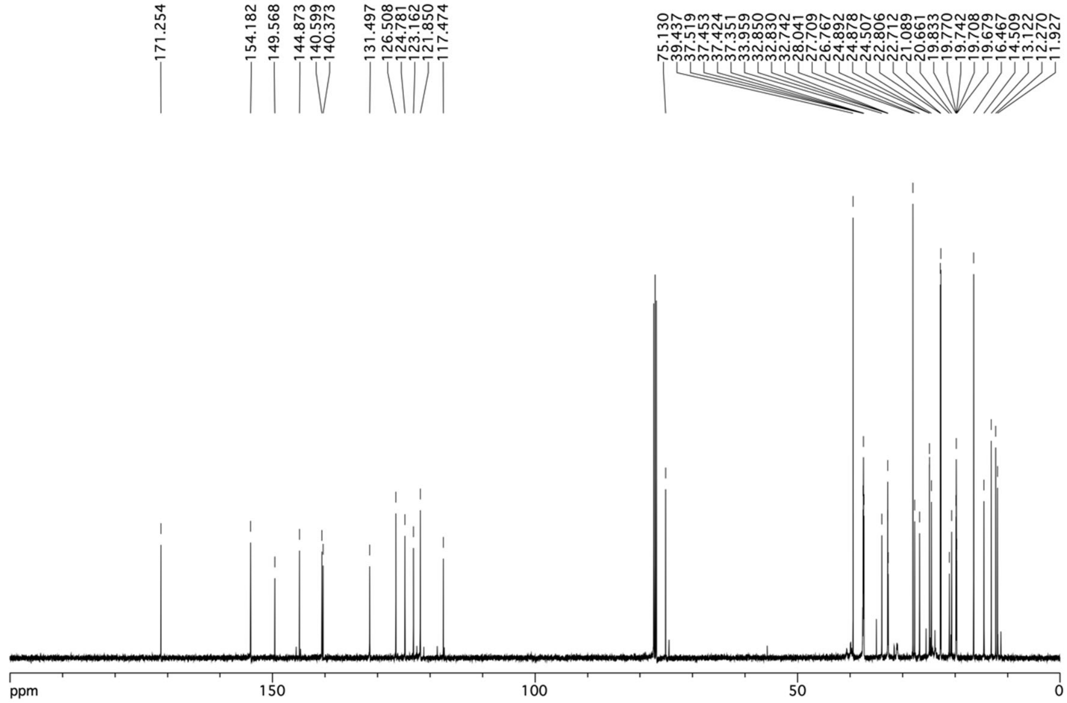

Figure S3. ^{13}C NMR spectrum of BDP-Toco.

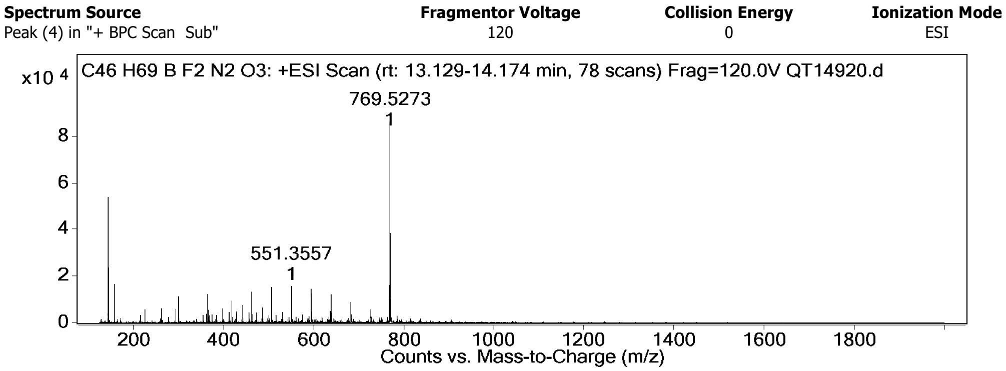

Figure S4. HRMS spectrum of BDP-Toco.

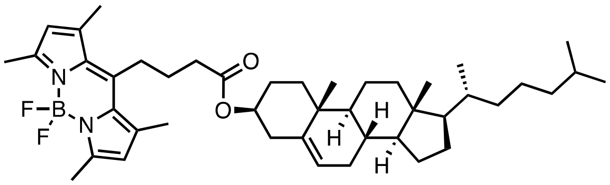

BDP-Chol

Figure S5. BDP-Chol.

To a solution of BDP-COOH (100 mg, 0.299 mmol, 1 eq) in DCM (3 mL) under Ar atm, Cholesterol (193 mg, 0.449 mmol, 1.5 eq) was added, followed by DCC (74 mg, 0.359 mmol, 1.2 eq) and DMAP (7.3 mg, 0.060 mmol, 0.2 eq). The reaction mixture was allowed to stir for 0.5 h. Then, the product was extracted with HCl (1 M) and washed two times with saturated Na_2CO_3. The organic phase was dried over anhydrous $MgSO_4$, filtrated and concentrated. The crude was purified by chromatography column on silica gel using DCM/Heptane (1:1) to obtain 143 mg of **BCP-Chol** (Yield = 68%) as an orange solid. Rf = 0.55 (DCM/Heptane, 1:1). ^{1}H-NMR (400 MHz, $CDCl_3$): δ 6.07 (s, 2H, H-β BODIPY), 5.40 (dd, *J* = 3.4, 1.3 Hz, 1H, CH cholest), 4.65 (dtd, *J* = 11.9, 8.1, 3.7 Hz, 1H, CH cholest), 3.00 (dt, *J* = 7.7, 4.3 Hz, 2H, CH_2-CO), 2.54 (s, 6H, 2 CH_3 BDP), 2.48 (t, *J* = 7.2 Hz, 2H, CH_2 BDP), 2.44 (s, 6H, 2 CH_3 BDP), 2.34 (d, *J* = 7.6 Hz, 2H, CH_2 Cholest), 2.05–1.83 (m, 7H, CH cholest), 1.63–0.93 (m, 28H, Cholest), 0.89 (dd, *J* = 6.6, 1.8 Hz, 6H, 2CH_3 Cholest), 0.70 (s, 3H, CH_3 Cholest). 13-C NMR (126 MHz; CDCl3): δ 171.9 (CO), 154.1, 145.1, 140.4, 139.5, 131.4, 122.8, 121.8, 74.3, 56.7, 56.1, 50.0, 42.3, 39.72, 39.53, 38.2, 37.0, 36.6, 36.2, 35.8, 34.7, 31.90, 31.85, 28.2, 28.0, 27.8, 27.5, 26.9, 24.3, 23.8, 22.8, 22.6, 21.0, 19.3, 18.7, 16.4, 14.5, 11.9. HMRS (ESI+) calculated for $C_{44}H_{65}BF_2N_2O_2$ [M^+]: 702.5107, found 702.5101.

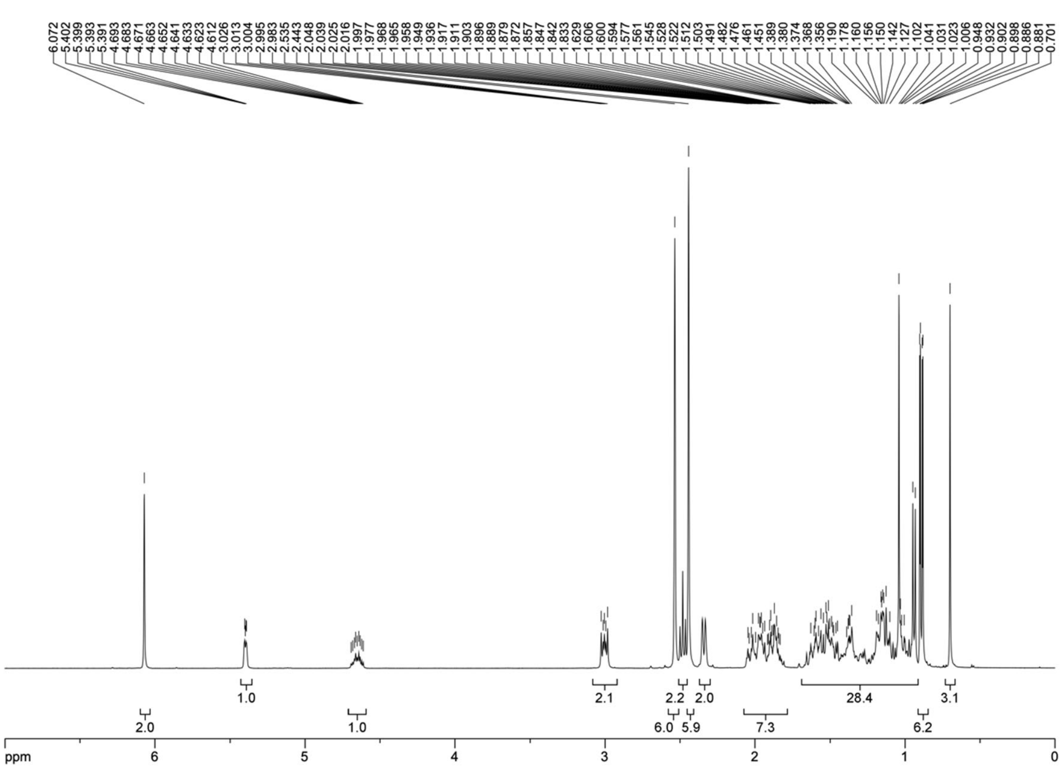

Figure S6. ^{1}H NMR spectrum of BDP-Chol.

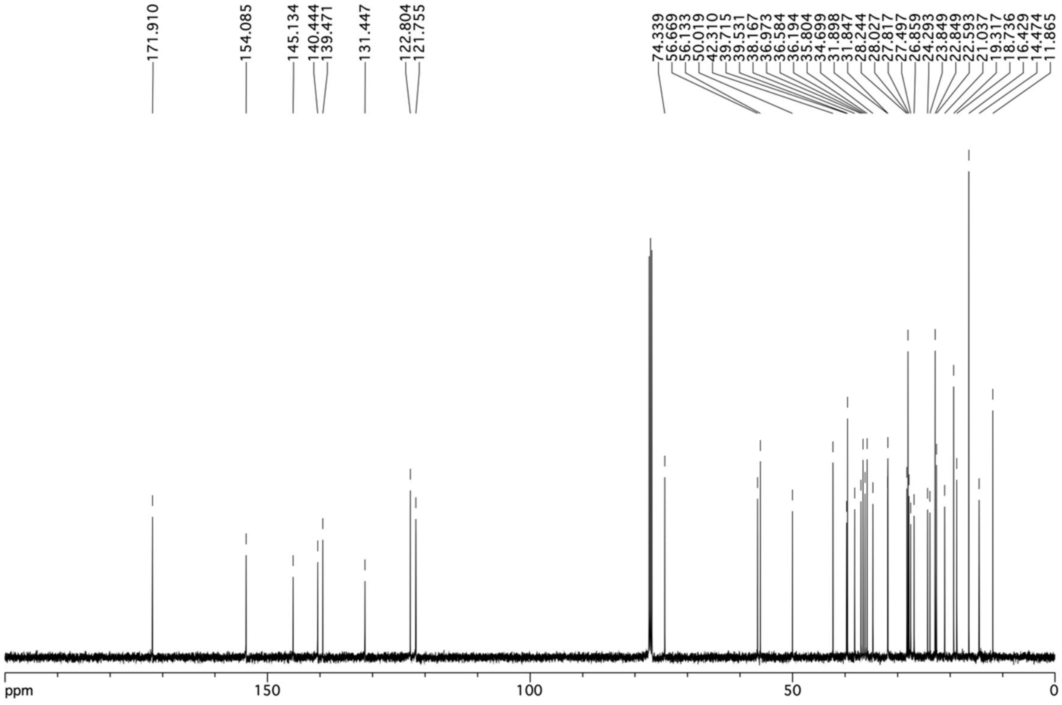

Figure S7. ^{13}C NMR spectrum of BDP-Chol.

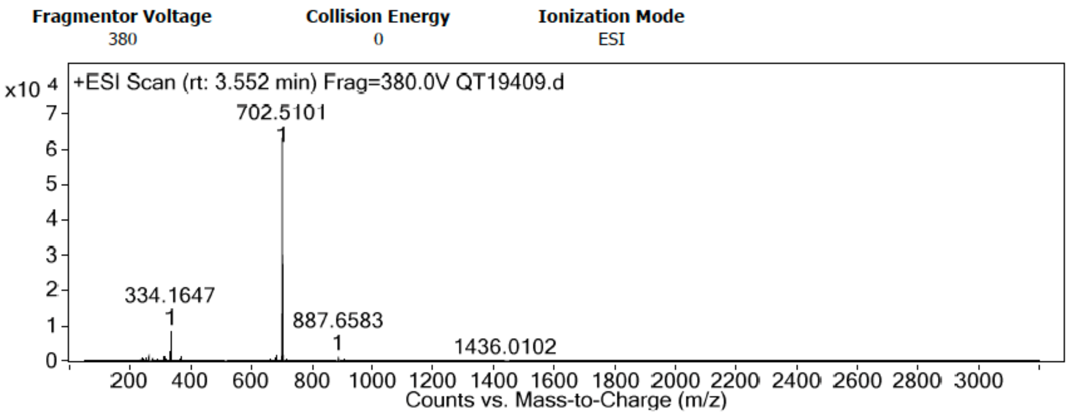

Figure S8. HRMS spectrum of BDP-Chol.

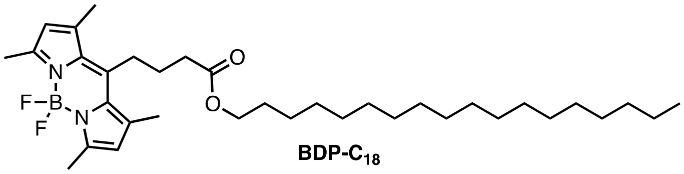

Figure S9. BDP-C_{18}.

To a solution of acid BDP-COOH (150 mg, 0.449 mmol, 1 eq) in DCM (3 mL) under Ar atm, stearyl alcohol (182 mg, 0.674 mmol, 1.5 eq) was added, followed by DCC (111 mg, 0.539 mmol, 1.2 eq) and DMAP (11 mg, 0.090 mmol, 0.2 eq). The reaction mixture was allowed to stir for 0.5 h. Then, the product was extracted with HCl (1 M) and washed two times with saturated Na_2CO_3. The organic phase was dried over anhydrous $MgSO_4$, filtrated and concentrated. The crude was purified by chromatography column on silica gel using DCM/Heptane (1:1) to obtain 123 mg of **BCP-C_{18}** (Yield = 22%) as an orange solid. Rf = 0.85 (DCM/Heptane, 1:1). ^{1}H-NMR (400 MHz, $CDCl_3$): δ 6.08 (s, 2H, H-β BODIPY), 4.10 (t, *J* = 6.8 Hz, 2H, OCH_2), 3.04–3.00 (m, 2H, CH_2-CO), 2.54–2.49 (m, 8H, 2 CH_3 BDP, CH_2), 2.45 (s, 6H, 2 CH_3 BDP), 1.97 (t, *J* = 8.2 Hz, 2H, CH_2), 1.67–1.63 (m, 2H, CH_2), 1.29 (m, 30H, 15 CH_2), 0.91 (t, *J* = 6.5 Hz, 3H, CH_3). ^{13}C NMR (100 MHz; $CDCl_3$): δ 172.6, 154.1, 145.0, 140.4, 131.4, 121.8, 64.9, 34.4, 31.9, 29.70, 29.68, 29.66, 29.65, 29.58, 29.52, 29.36, 29.26, 28.6, 27.5, 26.8, 25.9, 22.7, 16.3, 14.48, 14.46, 14.43, 14.1. HRMS (ES$^+$), for $C_{35}H_{57}BF_2N_2O_2Na$ $[M+Na]^+$ 609.4379, found 609.4385.

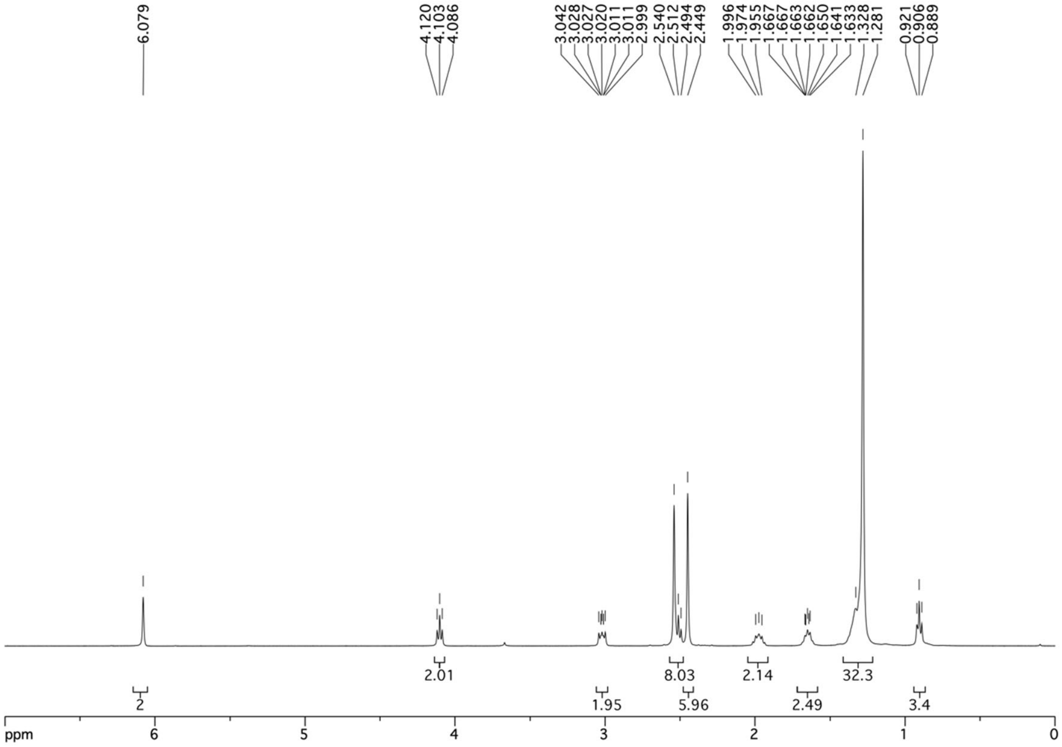

Figure S10. ^{1}H NMR spectrum of BDP-C_{18}.

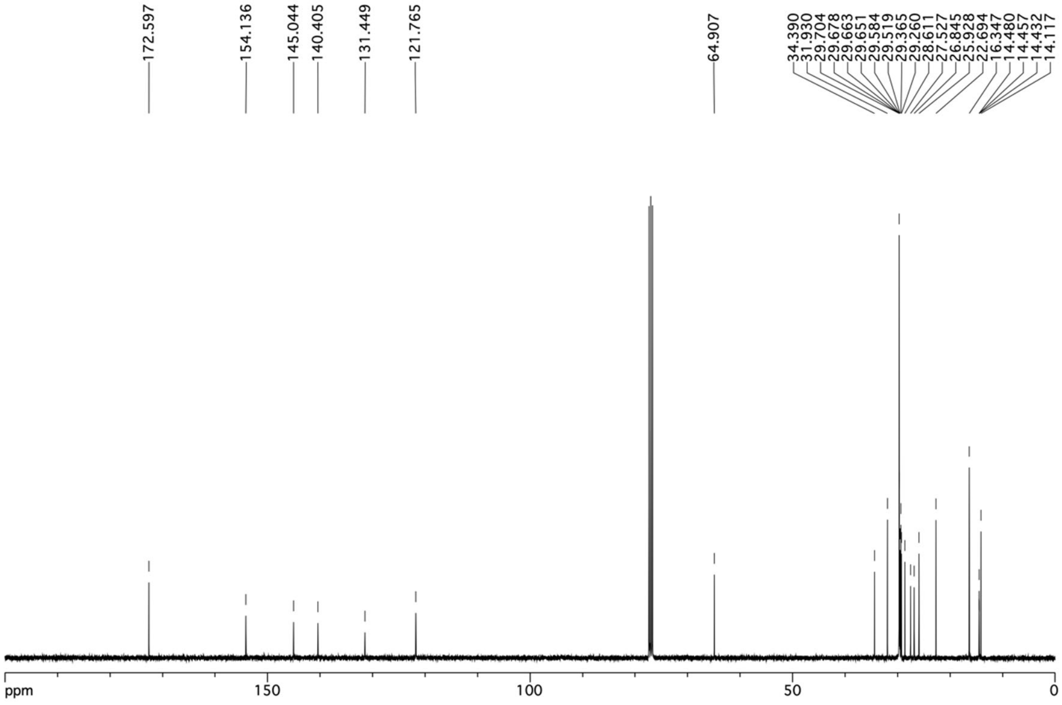

Figure S11. ^{13}C NMR spectrum of BDP-C_{18}.

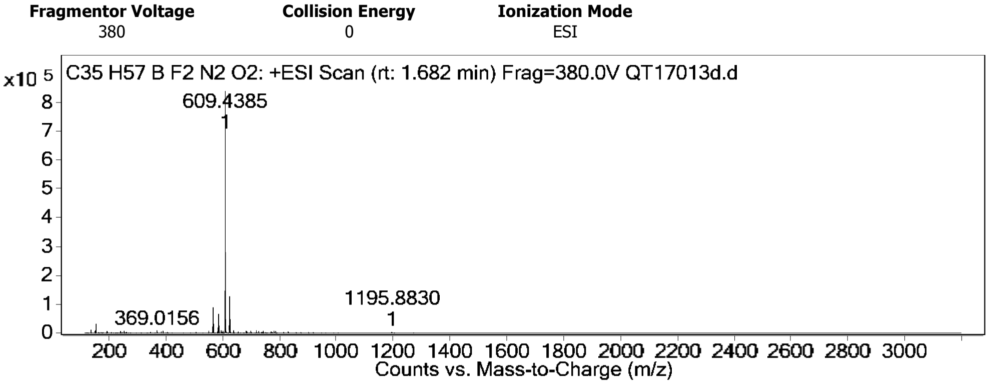

Figure S12. HRMS spectrum of BDP-C_{18}.

2. Calculation for the estimation of the individual brightness of NEs:

Considering VEA-based NEs loaded with 10 wt % of BDP-Toco.
Avogadro number: $N = 6.02\ 10^{23}\ mol^{-1}$
Molecular weight of the dye: $M = 746.8\ g.mol^{-1}$

3. Concentration of dye in oil:

10 wt % : 10 mg in 100 mg oil
Density of VEA: $d = 0.953\ g.mL^{-1}$
Volume: $V = m/d = 100/0.953 = 0.1049$ mL
Molar concentration: $C = n/V = m/(V \times M) = 10/(0.1049 \times 746.8) = 127.6$ mM

4. Volume of a 40 nm diameter NEs:

Volume: $V = 4/3 \times \pi \times r^3 = 4/3 \times 3.14 \times 20^3 = 33{,}493\ nm^3 = 3.349\ 10^{-20}$ L

5. Number of mol of dye in the NE:

Number of mol: $n = C \times V = 127.6\ 10^{-3} \times 3.349\ 10^{-20} = 427.3\ 10^{-23}$ mol

6. Number of dyes in the NE:

Number of dyes: $n_d = N \times n = 6.02\ 10^{23} \times 427.3\ 10^{-23} = 2572$ dyes

7. Brightness of an individual particle:

Brightness: $B = n_d \times \varepsilon \times \phi_{at\ 10wt\ \%} = 2572 \times 80{,}000 \times 0.22 = \sim 45.27\ 10^6\ M^{-1}.cm^{-1}$

8. Additional spectra

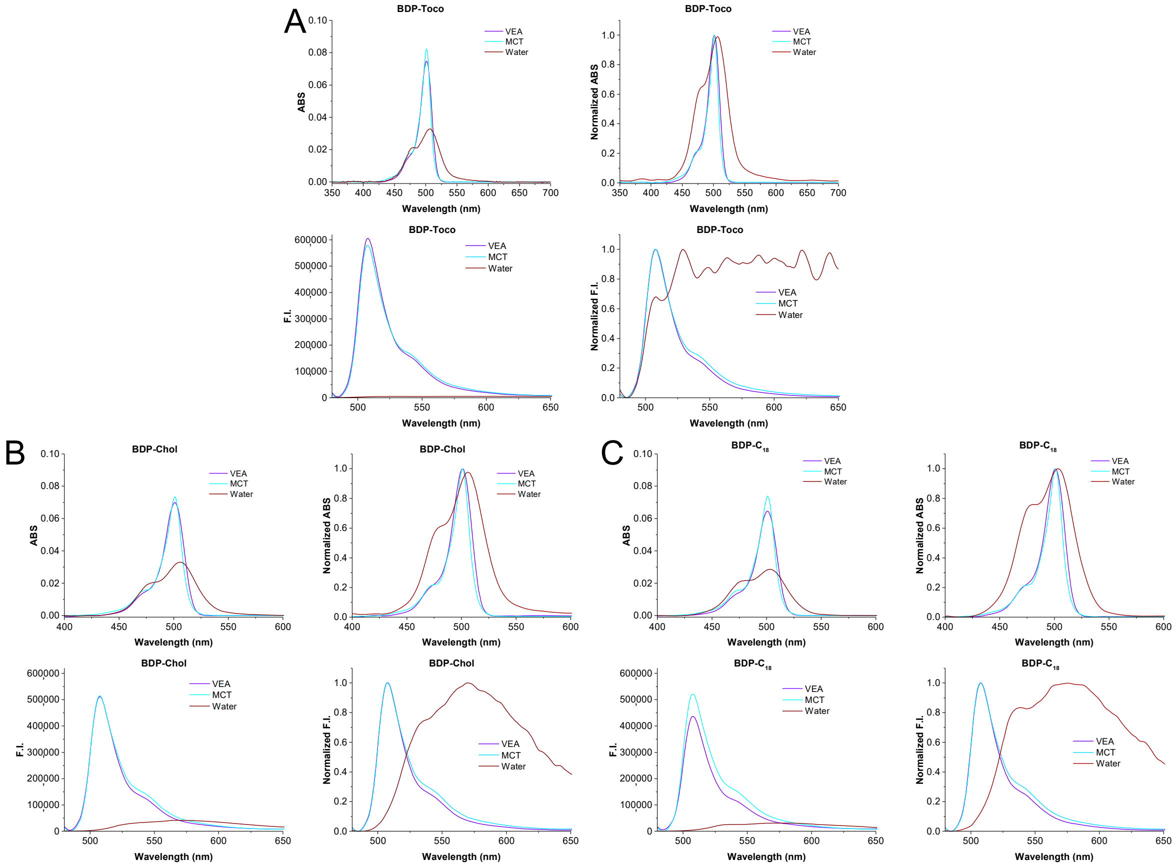

Figure S13. Non-normalized (**left**) and normalized (**right**) absorption (**top**) and emission (**bottom**) spectra of BDP-Toco (**A**), BDP-Chol (**B**) and BDP-C_{18} (**C**) in oils and water at 1 μM. Excitation wavelength was 470 nm.

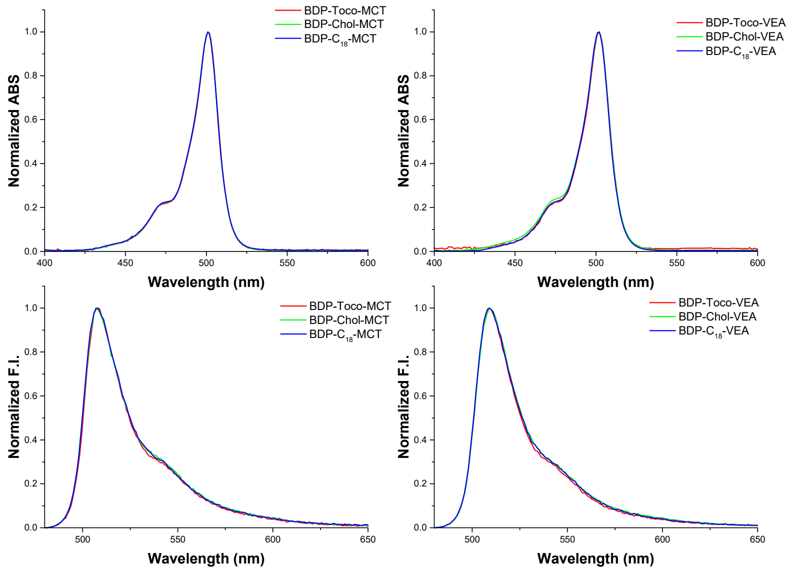

Figure S14. Normalized absorption (**top**) and emission (**bottom**) spectra of BDP-loaded NEs in MCT (**left**) and VEA (**right**) with a loading percentage of 1wt% in oil. Excitation wavelength was 470 nm.

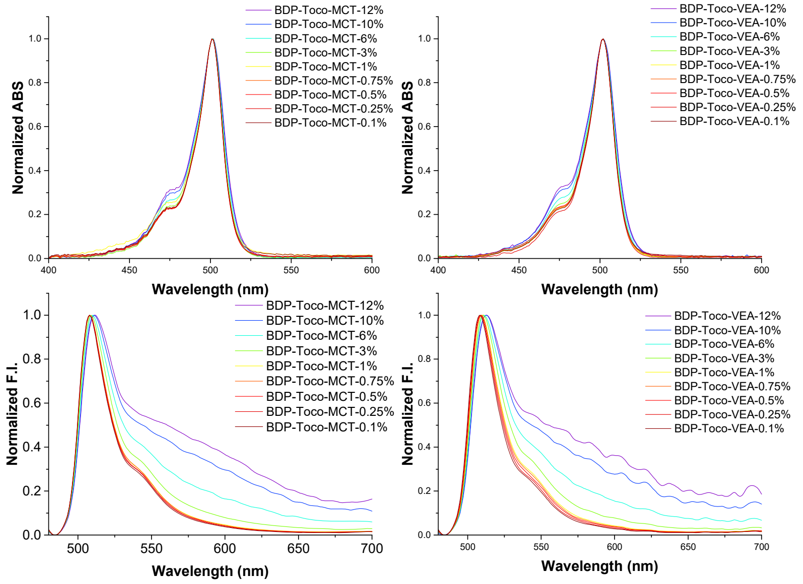

Figure S15. Normalized absorption (**top**) and emission (**bottom**) spectra of BDP-Toco NEs in MCT (**left**) and VEA (**right**) with an increase of loading percentage from 1% to 12% in oil. Excitation wavelength was 470 nm.
